# Spinal Cord Blood Flow after Ischemic Preconditioning in a Rat Model of Spinal Cord Ischemia

**DOI:** 10.1100/tsw.2004.186

**Published:** 2004-10-22

**Authors:** David Zvara, James M. Zboyovski, Dwight D. Deal, Jason C. Vernon, David M. Colonna

**Affiliations:** Department of Anesthesiology, Wake Forest University School of Medicine, Winston Salem, NC, USA

**Keywords:** spinal cord ischemia, ischemia and reperfusion, spinal cord blood flow

## Abstract

Spinal cord blood flow after ischemic preconditioning is poorly characterized. This study is designed to evaluate spinal cord blood flow patterns in animals after acute ischemic preconditioning. Experiment 1: After a laminectomy and placement of a laser Doppler probe over the lumbar spinal cord to measure spinal cord blood flow, 16 male Sprague-Dawley rats were randomized into two groups: ischemic preconditioning (IPC, n = 8), and control (CTRL, n = 8). Rats in the CTRL and the IPC groups were subjected to 12 min of ischemia directly followed by 60 min of reperfusion. IPC rats received 3 min of IPC and 30 min of reperfusion prior to the 12-min insult period. Experiment 2: After instrumentation, the rats were randomized into three groups: control (CTRL, n = 7), ischemic preconditioning (IPC, n = 7), and time control (TC, n = 4). Rats in the CTRL and the IPC groups were subjected to the same ischemia and reperfusion protocol as above. The TC group was anesthetized for the same time period as the CTRL and the IPC groups, but had no ischemic intervention. Microspheres were injected at baseline and at 15 and 60 min into the final reperfusion. All rats were euthanized and tissue harvested for spinal cord blood flow analysis. In Experiment 1, there was a slight, significant difference in spinal cord blood flow during the ischemic period; however, this difference soon disappeared during reperfusion. In experiment 2, there was no difference in blood flow at any experimental time. The results of these experiments demonstrate that IPC slightly enhances blood flow to the spinal cord during ischemia; however, this effect is not sustained during the reperfusion period.

## INTRODUCTION

Ischemic preconditioning (IPC) is an endogenous cellular protective mechanism in which a brief, noninjurious ischemic episode and reperfusion precede a longer ischemic insult yielding the tissue more resistant to a subsequent hypoxic insult. This physiologic protective mechanism was first defined by Murry[1] in heart tissue. Since this initial report, IPC has been found in multiple organs including the brain[2], spinal cord[3,4], and kidney[5].

Several investigators have reported benefit of IPC in protecting the spinal cord after transient aortic occlusion. Munyao and colleagues[6] report improved hindlimb function in rabbits after 12.5 min of preconditioning followed either 24 or 48 h later by 30 min of aortic occlusion. In a similar model, Abraham et al.[3] reported a reduced incidence of paraplegia in rats with either 2 or 5 min of IPC 48 h before a 10-min aortic occlusion. Matsuyama and colleagues[7] reported reduced neurologic injury with IPC and elevated heat shock protein (HSP) levels in ischemic preconditioned dogs compared to controls. In a similar report, Sakurai and colleagues[8] reported spinal cord protection 48 h after IPC in a rabbit model of aortic occlusion. HSP 70 messenger RNA was significantly elevated in the IPC group.

In contrast to these models of delayed IPC, Fan et al. first reported neurologic protection in a model of acute IPC[9]. Neurologic recovery was assessed by spinal cord evoked potentials. In this model, 5 min of IPC increased regional spinal cord blood flow. Zvara et al.[4] reported spinal cord protection in a similar model of acute IPC protection. In this report, neurologic injury was assessed by physical examination 48 h after injury. IPC rats demonstrated significantly improved neurologic function and reduced evidence of spinal cord injury on postmortem histology. Other investigators have reported similar neurologic protection soon after the IPC event[2,10].

There is likely more than one mechanism of action for the beneficial effects of IPC in spinal cord protection after transient aortic occlusion. In models of delayed protection, HSPs may play some role[7,8]; whereas in models of acute protection, the mechanism is much less clear. Miyamoto and Miyamoto suggest that acute IPC may be due to an outpouring of adenosine during the brief IPC episode with subsequent release of the protective neuroinhibitory amino acid taurine[11]. Fan et al.[9] measured regional catecholamines and their metabolites demonstrating increased levels after ischemia associated with diminished regional spinal cord blood flow. Data from Zvara[4] and colleagues show alterations consistent with changes in regional spinal cord blood flow. In their report, the changes in both heart rate (HR) and blood pressure are consistent with adenosine release immediately after IPC compared to control animals. In view of this possible mechanism of protection, there may be associated changes of ischemic and reperfusion spinal cord blood flow patterns in ischemic preconditioned animals as compared to controls. We hypothesized that IPC 30 min prior to an ischemic reperfusion injury alters ischemic and reperfusion blood flow patterns. These altered blood flow patterns may, in part, explain the benefit seen after acute IPC in a rat model of transient spinal cord ischemia.

## METHODS

The Institutional Animal Care and Use Committee of Wake Forest University School of Medicine, Winston Salem, NC, approved all the surgical and testing procedures. The animal protocol was conducted in accordance with the “Guide for the Care and Use of Laboratory Animals” published by the National Institutes of Health (NIH publication 85-23, revised 1985).

Male Sprague-Dawley rats weighing 467 ± 75 g were used in this experiment. The rats were provided with standard rat chow and water *ad libitum*. All rats were neurologically intact prior to anesthesia and instrumentation.

## Surgical Procedure

The rats were anesthetized with 1.5% halothane in a 45% oxygen and air mixture. They were allowed to breathe spontaneously with a nonsealing facemask device. A temperature probe (YSI Inc., Yellow Springs, OH, model 402) was inserted into the rectum and body temperature was measured and maintained between 37.5EC and 38.5EC with a circulating warm water (37.5EC) underbody heating pad. The tail artery was cannulated with a PE-50 polyethylene catheter to obtain arterial blood samples and to monitor mean distal aortic blood pressure (MDAP). The left femoral artery was isolated, and a Fogarty 2F balloon-tipped catheter (Baxter, Santa Ana, CA) was introduced for later advancement into the descending thoracic aorta. The left internal carotid artery was cannulated with a 20-gauge catheter for measurement of mean proximal aortic pressure (MPAP). The carotid artery cannula was connected to a heated blood-collection circuit (37.5EC) that included an 88-cm vertical column of heparinized normal saline solution (1 U/ml). When the aorta was occluded, proximal aortic blood was allowed to bleed into the heparinized column, maintaining MPAP at 60 ± 2 mmHg. Mean proximal aortic pressure, mean distal aortic pressure, and temperature were recorded at 1-min intervals by a PC-based data acquisition system (Micro-Med, Louisville, KY). After instrumentation, all rats were given 200 units of heparin sodium. Arterial blood gas sampling was performed prior to aortic occlusion (baseline) and at the end of the procedure just prior to euthanization.

To induce spinal cord ischemia, the Fogarty 2F catheter in the left femoral artery was advanced retrograde into the descending thoracic aorta 10.5 cm from the femoral arteriotomy so that the tip of the catheter balloon lay just caudal to the left subclavian artery. This distance was confirmed at postmortem examination of three rats sacrificed for catheter placement measurements. During the experimental protocol, the catheter was inflated with 0.05 ml of saline solution, and aortic occlusion was confirmed by reduction in MDAP.

### *Experiment 1: Spinal Cord Blood Flow Measurements Using Laser Doppler Flowmetry*

After instrumentation, all rats were placed prone and a surgical incision was made along the lumbar vertebral column. A laminectomy at L1 was performed to expose the spinal cord. A laser Doppler flow probe (PF 403, Perimed AB, Stockholm, Sweden) was fixed to a manipulator (KOPF7, Tujunga, CA) and positioned <1 mm from the exposed spinal cord. Proper positioning of the flow probe was confirmed by surgical microscope throughout the procedure. A Periflux Master 4001-2 laser Doppler flowmeter was used to monitor changes in blood flow. Blood flow was measured in terms of perfusion units (1-999) and is reported as a percent change from baseline.

Rats were randomized into two groups: IPC (n = 8), and control (CTRL, n = 8). Rats in the CTRL and the IPC groups were subjected to 12 min of ischemia directly followed by 60 min of monitored reperfusion. IPC rats received 3 min of IPC and 30 min of reperfusion prior to the 12-min insult period. At the end of the ischemic periods, the vented blood from the carotid artery cannula was reinfused over 60 sec. Laser Doppler flowmetry data were continuously recorded for 60 min during reperfusion. At the completion of data collection all rats were euthanized.

### *Experiment 2: Spinal Cord Blood Flow Measurements Using Colored Microspheres*

In a second group of rats, spinal cord blood flow before ischemia and during reperfusion was evaluated using colored microspheres; red, coral, violet, or orange (15 ± 0.5 μm diameter, E-Z TRAC, Inc., North Hollywood, CA). After instrumentation, the rats were randomized into three groups: control (CTRL, n =

7), IPC (IPC, n = 7), and time control (TC, n = 4). Rats in the CTRL and the IPC groups were subjected to 12 min of ischemia directly followed by 60 min of monitored reperfusion. IPC rats received 3 min of IPC and 30 min of reperfusion directly following instrumentation and prior to the 12-min insult period. The TC group was anesthetized for the same time period as the CTRL and the IPC groups. Surgical procedures in the TC group were the same as the CTRL and IPC groups, but TC rats were never subjected to ischemic insult.

## Preparation and Injection of Colored Microspheres

Nonradioactive colored microspheres are cross-linked polystyrene-divinylbenzene microspheres that have been labeled with distinct colors: red, orange, coral, and violet. In this study, three random colors were used for each experiment. Vials obtained from the manufacturer contained $10^6$ microspheres/ml suspended in distilled water containing 0.05% Tween 80 and 0.01% thimerosal. For each experiment, the solution containing the microspheres was agitated mechanically and ultrasonically in a vortex mixer for 4 min. A femoral arterial PE-50 catheter was connected to a constant rate withdrawal pump just before each injection. The microspheres (0.2 ml = 2 H $10^5$) were injected over a period of 20 sec through a PE-50 polyethylene catheter temporarily positioned in the left ventricle via the right common carotid artery. The tubing was flushed immediately with 0.6 ml of 13.4 vol% Ficoll 70 as a fluid replacement. A 0.6-ml aliquot of 13.4 vol% solution produced the same oncotic pressure as does the amount of plasma protein in 1.0 ml of blood. The reference blood sample was withdrawn from the femoral catheter beginning 10 sec before the microsphere injection and continuing for 60 sec at a constant rate of 0.75 ml/min (Harvard Apparatus, model 55-2226, Southmatick, MA).

## Blood and Tissue Processing

Reference blood samples and individual tissue samples were centrifuged, and the sediment was treated with a series of reagents prescribed by the manufacturer (Interactive Medical Technologies, Ltd., Los Angeles, CA) to lyse the red blood cells and digest tissue. The total number of microspheres per reference sample and tissue sample was determined by examining an aliquot (~18 µl) of the final microsphere suspension (<30 µl). Using a computer-assisted microscopic imaging device (TRACKER 1000; Interactive Medical Technologies, Ltd., Los Angeles, CA), this aliquot was placed in a Fuchs-Rosentahl hemocytometer and 240 frames were imaged and recorded. The total number of each color microsphere imaged was converted to the total number for each individual blood and tissue sample by the formula:

$$Q_m = (C_m \text{ H } Q_r)/C_r$$

where $Q_m$ is the regional blood flow per gram (ml · min$^{-1}$ · g$^{-1}$), $C_m$ is the microsphere count per gram of tissue, $Q_r$ is the withdrawal rate of the reference blood sample (ml/min), and $C_r$ is the microsphere count in the reference blood sample.

## Statistical Analysis

HR, MPAP, MDAP, and temperature were analyzed using two-way repeated-measures analysis of variance. For all statistical analysis a *p* value of <0.05 was considered significant. Data are expressed as the mean ± the standard error of the mean unless otherwise indicated.

## RESULTS

Experiment 1: MPAP, MDAP, HR, and temperature were not different between IPC group and CTRL groups during the experimental protocol (Table 1). Laser Doppler flow data are shown in Fig. 1. Both groups exhibited an expected drop in lumbar blood flow during ischemia, which promptly returned on initiation of reperfusion. There is a slight difference in spinal cord blood flow during the later half of ischemia in the IPC animals. There was an initial hyperemia associated with early reperfusion that was not different between groups.

**TABLE 1**
**Laser Doppler Hemodynamics**

| | | Baseline A | DIPC | Baseline B | ISC | REP15 | REP60 |
|---|---|---|---|---|---|---|---|
| Temp (EC) | CTRL | 37.9 ± 0.5 | 37.9 ± 0.4 | 37.8 ± 0.3 | 37.6 ± 0.2 | 37.5 ± 0.2 | 37.8 ± 0.3 |
| | IPC | 37.8 ± 0.5 | 37.6 ± 0.5 | 37.7 ± 0.3 | 37.3 ± 0.3 | 37.4 ± 0.3 | 37.8 ± 0.3 |
| HR (bpm) | CTRL | 285 ± 17 | 298 ± 17 | 297 ± 27 | 225 ± 44 | 266 ± 10 | 275 ± 13 |
| | IPC | 312 ± 42 | 226 ± 62 | 286 ± 38 | 207 ± 41 | 255 ± 25 | 274 ± 24 |
| MDAP (mmHg) | CTRL | 67.4 ± 9.2 | 70.0 ± 11.2 | 64.9 ± 5.0 | 0.8 ± 1.8 | 77.6 ± 11.6 | 62.1 ± 7.5 |
| | IPC | 70.5 ± 4.5 | 3.3 ± 4.1* | 64.5 ± 8.9 | 2.0 ± 2.7 | 75.7 ± 13.9 | 57.8 ± 5.1 |
| MPAP (mmHg) | CTRL | 84.9 ± 11.2 | 85.1 ± 9.0 | 74.2 ± 6.6 | 61.0 ± 2.4 | 92.0 ± 11.5 | 73.0 ± 5.4 |
| | IPC | 82.4 ± 5.0 | 62.7 ± 2.9 | 74.2 ± 8.3 | 61.4 ± 3.0 | 91.3 ± 11.7 | 72.4 ± 8.1 |

*$p < 0.001$ compared to CTRL. Baseline A = values recorded prior to any ischemic or reperfusion event; DIPC = during ischemic preconditioning; Baseline B = values recorded just prior to the 12-min ischemic event; ISC = 12 min of ischemia; REP15 = 15 min into reperfusion; REP60 = 60 min into reperfusion; CTRL = vehicle group; IPC = ischemic preconditioned group.

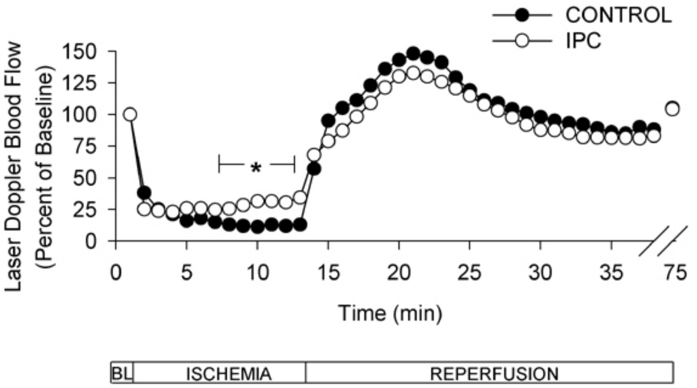

**FIGURE 1**. Percent change in laser Doppler blood flow for baseline at the lumbar spinal cord. * = significant difference between groups in laser Doppler blood flow, $p < 0.05$.

Experiment 2: Hemodynamics are shown in Table 2. Fig. 2 demonstrates the change in blood flow in the cervical, thoracic, and lumbar spinal cord in control, IPC, and time control animals at reperfusion 15 and reperfusion 60 compared to baseline. There is no difference between IPC and control animals in blood flow during these reperfusion intervals.

**TABLE 2**
**Microsphere Blood Flow Hemodynamics**

| | | Baseline A | DIPC | Baseline B | ISC | REP15 | REP60 |
|---|---|---|---|---|---|---|---|
| Temp | CTRL | 37.3 ± 0.4 | 37.3 ± 0.4 | 37.4 ± 0.3 | 37.4 ± 0.2 | 37.1 ± 0.5 | 37.4 ± 0.3 |
| (EC) | IPC | 37.3 ± 0.4 | 37.2 ± 0.4 | 37.3 ± 0.4 | 37.3 ± 0.4 | 36.6 ± 0.3 | 37.3 ± 0.4 |
| | TC | 37.4 ± 0.3 | 37.4 ± 0.4 | 37.9 ± 0.4 | 37.8 ± 0.4 | 37.6 ± 0.5 | 37.8 ± 0.2 |
| HR | CTRL | 315 ± 23 | 312 ± 22 | 285 ± 18 | NA | 268 ± 26 | 304 ± 23 |
| (bpm) | IPC | 315 ± 26 | NA | 284 ± 24 | NA | 238 ± 26 | 292 ± 29 |
| | TC | 348 ± 17 | 351 ±20 | 327 ± 30 | 333 ± 24 | 328 ± 43 | 321 ± 29 |
| MDAP | CTRL | 84.4 ± 6.4 | 82.5 ± 6.7 | 78.2 ± 9.0 | 10.3 ± 2.7 | 71.9 ± 12.6 | 70.2 ± 11.3 |
| (mmHg) | IPC | 82.5 ± 11.5 | 13.5 ± 2.1* | 71.3 ± 14.4H | 9.1 ± 2.0 | 81.5 ± 18.4 | 60.8 ± 9.9 |
| | TC | 90.2 ± 5.1 | 90.2 ± 6.6 | 86.9 ± 5.8 | 85.5 ± 5.3I | 85.7 ± 4.5 | 87.6 ± 4.3' |

**p* < 0.001 compared to CTRL and TC; H*p* = 0.026 compared to TC; I*p* < 0.001 compared to CTRL and IPC; '*p* < 0.001 compared to IPC; *p* = 0.004 compared to CTRL; DIPC = during ischemic preconditioning; CTRL = control group; IPC = ischemic preconditioned group; TC = time control group; NA = not available (HR not available secondary to cannula manipulation during ischemia allowing microsphere injection).

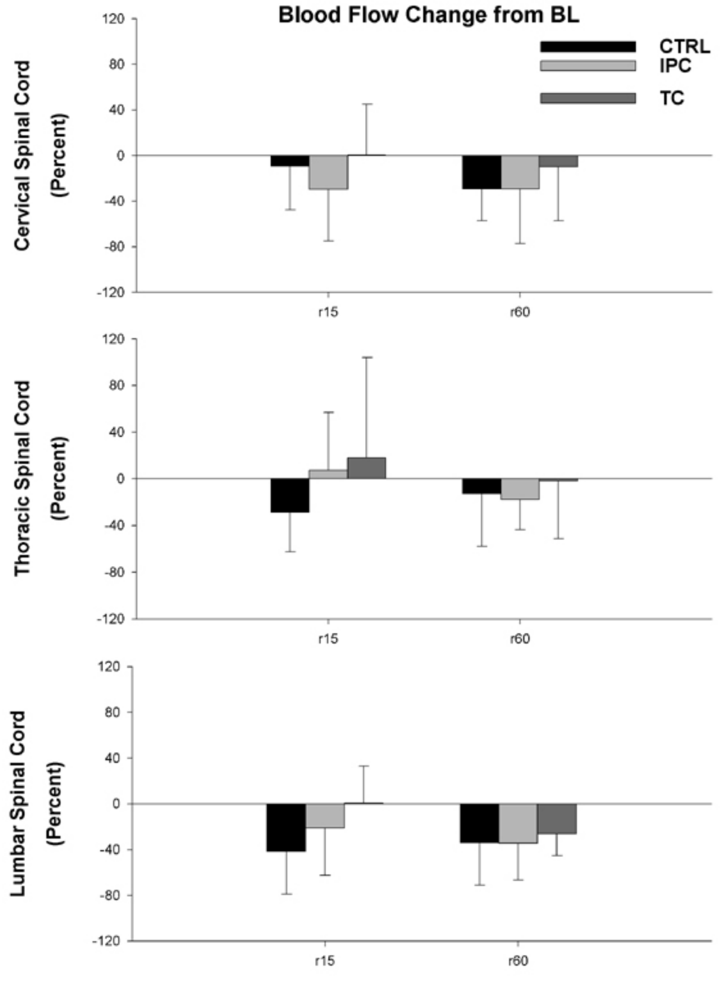

**FIGURE 2**. Change in microsphere calculated blood flow compared to baseline in control (CTRL), ischemic preconditioned (IPC), and time control animals (TC). r15 = 15 min into reperfusion; r60 = 60 min into reperfusion.

## DISCUSSION

The results of these experiments demonstrate that IPC slightly enhances blood flow to the spinal cord during ischemia; however, this effect is not sustained during the reperfusion period.

Numerous reports have documented the various “time windows of protection” associated with IPC of neuronal tissue. Much research has been done on the delayed window of protection, e.g., neuronal protection that is manifest 12, 24, or 48 h after the preconditioning event[3,6,7,8,12]. A second window of protection, one occurring much earlier after the preconditioning event, is also present in neuronal tissue. In a model of global cerebral ischemia, Perez-Pinzon[10] demonstrated improved neurologic protection 30 min after a preconditioning event. Reshef and colleagues[2] found similar results with an even shorter time of reperfusion between the preconditioning event and the subsequent ischemic event. In their model of neuronal cultures, IPC, or exposure to the A1 receptor agonist N6-(R)-phenylisopropyladenosine (R-PIA), exhibited protection as early as 10 min after the preconditioning event. In a rabbit model of acute ischemic preconditioning, Fan et al.[9] demonstrated reduced spinal cord injury by evoked potential monitoring. Zvara and colleagues[4] demonstrated improved neurologic outcome and spinal cord histology with the preconditioning event separated from the ischemic insult by 30 min. As with the models of delayed preconditioning, the mechanisms of acute protection remain unknown. Indirect evidence from Zvara et al.[4] and, more directly, from Reshef et al.[2] and Fan et al.[9] suggests a role for vasoactive compounds.

Adenosine is intimately involved in the mechanism of IPC. Adenosine is a very short-acting substance released from the ischemia induced degradation product of ATP. During ischemia, adenosine activates the A1 receptor on the cell membrane initiating a series of intracellular events including activation of G-proteins, phospholipase C, protein kinase C, and ATP-sensitive potassium channels, and release of neurotransmitters[13,14,15]. The specific roles that adenosine, ATP sensitive potassium channels, or other agents may play in models of acute preconditioning remain unknown. Because adenosine may play a role in models of acute ischemia, there may be significant differences in regional blood flow patterns in ischemic preconditioned animals as compared to control animals. Similarly, the role of catecholamines and their metabolites after ischemic injury may play a significant role.

In this experiment, blood flow patterns after an IPC event were evaluated. In the first experiment, laser Doppler analysis was used to characterize blood flow during the ischemic and second reperfusion interval. Consistent with the release of adenosine, or perhaps, as suggested by Miyamoto and Miyamoto[11], the adenosine mediated release of taurine, there was evidence of enhanced blood flow in the IPC animals. This enhanced blood flow was of limited duration. The differences between groups disappeared during reperfusion. In Experiment 2, there was no evidence of differing blood flow patterns during reperfusion between IPC animals and control. Given the minimal changes in Experiment 1, and the results of Experiment 2, we conclude that blood flow patterns are not substantively different in IPC animals. This conclusion does not negate a role for adenosine, catecholamines, or other agents. Rather, the conclusion suggests that whatever neuroprotection is afforded the animal is likely via some other mechanism than spinal cord blood flow alteration during IPC.

This paper is limited by the relatively narrow focus of the experimental design. No attempt was made to seek other mechanisms of IPC protection. HSP was not assayed, nor were adenosine or catecholamines. ATP-sensitive potassium channels were not evaluated. An additional limitation is that this experiment does not evaluate the rats for postintervention recovery. Unfortunately, the experimental design did not allow animal survival. The groups differ in intervention and based on previous work by this laboratory and others, we assumed that this intervention would lead to differing outcomes if the rats were allowed to survive. Future studies may address these issues.

## BIOSKETCH

**David A. Zvara** is an Associate Professor in the Department of Anesthesiology at Wake Forest University School of Medicine. He received his B.S. and M.D. degrees at Ohio State University and is a Fellow of the American Heart Association.
